# Research and Nonresearch Industry Payments for COVID-19 mRNA Vaccines in the United States

**DOI:** 10.31662/jmaj.2022-0210

**Published:** 2023-03-06

**Authors:** Anju Murayama, Sae Kamamoto, Hiroaki Saito, Tetsuya Tanimoto, Akihiko Ozaki

**Affiliations:** 1 Medical Governance Research Institute, Tokyo, Japan; 2 Tohoku University School of Medicine, Sendai, Japan; 3 Hamamatsu University School of Medicine, Hamamatsu, Japan; 4 Department of Gastroenterology, Sendai Kousei Hospital, Sendai, Japan; 5 Department of Internal Medicine, Navitas Clinic Tachikawa, Tokyo, Japan; 6 Department of Breast and Thyroid Surgery, Jyoban Hospital of Tokiwa Foundation, Iwaki, Japan

**Keywords:** COVID-19, physician payment, Open Payments Database, SARS-CoV-2, vaccine, industry payment

While vaccines developed for the coronavirus diseases 2019 (COVID-19) are highly effective ^(1)^, only 67.4% and 48.5% of US citizens received full vaccination and additional first booster vaccination, respectively, as of August 30, 2022 ^(2)^. One possible explanation for vaccine hesitancy is concern over financial relationships between healthcare professionals (HCPs) and organizations (HCOs) and pharmaceutical companies manufacturing COVID-19 vaccines ^(3), (4)^. However, the information is lacking on these financial relationships in the US. This study aimed to evaluate the magnitude of payments made by pharmaceutical companies manufacturing two COVID-19 mRNA vaccines to HCPs and HCOs, using the Open Payments Database in 2021.

This cross-sectional study included all research and nonresearch payments related to two COVID-19 mRNA vaccines (COMIRNATY [or Pfizer-BioNTech COVID-19 Vaccine] from Pfizer and BioNTech and SPIKEVAX [or Moderna COVID-19 Vaccine] from Moderna) receiving the emergency use authorization by the US FDA as of December 31, 2021. We extracted all research and nonresearch (general) payments related to the COVID-19 vaccines being made to both HCPs and teaching hospitals from the Open Payments Database between January 2020 and December 2021.

The research and nonresearch payment data were descriptively analyzed. For research payments, payments per physician were calculated based on total amounts of payments divided by the number of principal investigators in a research payment. We defined principal investigators as physicians listed in the payment data as payment recipients when a research payment was made to a teaching hospital or a recipient noncovered by the Open Payments such as a research institute and university. This study was approved by the Ethics Committee of the Medical Governance Research Institute (approval number: MG2018-04-20200605; approval date: June 5, 2020). Informed consent was waived, as this study included only publicly available information.

Payments related to the COVID-19 mRNA vaccines in 2020 are not found, and the following analyses all accounted for the payments in 2021. In 2021, a total of $356,419,505.39 were made to 372 physicians, 2 nurse practitioners, and 4 teaching hospitals, which were all related to the Pfizer-BioNTech COVID-19 Vaccine or the Moderna COVID-19 Vaccine (Table 1). Of $356,419,505.39 industry payments, $355,630,760.16 equivalent to 99.8% of overall industry payments were made for research payments, and 69.4% ($246,874,732.49) of research payments were made for Pfizer-BioNTech COVID-19 Vaccine. Among the 22 research projects associated with the payment data, 21 research projects (95.5%) were available for their details, which were all clinical trials evaluating efficacy and safety of the COVID-19 vaccines. The median payments per payment recipient were $550,964.96 (interquartile range [IQR]: 63,058.98-1,436,790.37), while average payments were $1,039,856.02 (standard deviation [SD]: $1,383,929.03).

**Table 1.** Research and Nonresearch Payments Made to Healthcare Professionals and Healthcare Organizations By Pharmaceutical Companies Manufacturing COVID-19 Vaccines between 2020 and 2021.

Variables	Type of COVID-19 vaccines			Overall
	Pfizer-BioNTech COVID-19 Vaccine		Moderna COVID-19	
	BioNTech	Pfizer	Vaccine	
**Overall**				
Total payment amounts, $ (%)	4,252,074.58 (1.2)	242,966,738.08 (68.2)	109,200,692.69 (30.6)	356,419,505.35
Number of payment recipients, n	12 physicians	232 physicians	175 physicians	372 physicians
	1 teaching hospital	3 teaching hospitals	2 nurse practitioners	2 nurse practitioners
				4 teaching hospitals
**Research payments**				
Payments amounts, $ (%)	4,140,488.58 (1.2)	242,734,243.91 (68.3)	108,756,027.67 (30.6)	355,630,760.16 (99.8)
Number of payment recipients, n	12 physicians	224 physicians	150 physicians	340 physicians
		2 teaching hospitals		2 teaching hospitals
Median payments per recipient (IQR), $	198,674.75	612,737.36	321,262.67	550,964.96
	(36,380.81-490,409.02)	(94,859.41-1,498,176.54)	(56,720.25-1,153,151.65)	(63,058.98-1,436,790.37)
**Nonresearch payments**
Payment amounts, $ (%)	111,586 (14.1)	232,494.17 (29.5)	444,665.02 (56.4)	788,745.19 (0.2)
Number of payment recipients, n	1 teaching hospital	9 physicians	25 physicians	34 physicians
		3 teaching hospitals	2 nurse practitioners	2 nurse practitioners
				4 teaching hospitals
Median payments per recipient (IQR), $	7,800 (4,900-50,000)*		2,100 (1,600-3,575)	3,350 (1,637.5 7,850)
Nature of payments, $ (%)
Consulting	0 (0)	113,604.17 (48.9)	444,613 (100.0)	558,217.17 (70.8)
Royalty and license	111,586 (100.0)	100,000 (43.0)	0 (0)	211,586 (26.8)
Speaking compensation	0 (0)	18,890 (8.1)	0 (0)	18,890 (2.4)
Food and beverage	0 (0)	0 (0)	52.02 (0.01)	52.02 (0.01)

Abbreviation: IQR (interquartile range) * Median payments per recipient were calculated for Pfizer and BioNTech, as BioNTech made only one nonresearch payment in 2021. There was no record of research and nonresearch payments in 2020 in the Open Payments Database.

Meanwhile, nonresearch payments accounted for only $788,745.19 (0.2%). The most payment type was consulting fees, accounting for 70.8% ($558,217.17) of all nonresearch payments, followed by 26.8% ($311,586 for Pfizer-BioNTech COVID-19 Vaccine) for royalty. Moderna made one payment worth $52.02 in meal and beverage. Median payments were 3,350 (IQR: 1,637.5-7,850) per recipient.

This study suggests that almost all industry payments related to the COVID-19 vaccines were paid for research purposes where there are imminent needs for evaluating vaccine efficacy and safety in various important sub-populations, such as children and patients with other diseases after their approval. Only a few number of HCPs received financial incentive from pharmaceutical industries for the two COVID-19 mRNA vaccines, indicating that highly effective medical products need not intensive promotions even in critical situation such as the COVID-19 pandemic, as the vaccines have been promoted and distributed by official agencies, including the Centers for Disease Control and Prevention, the National Institute of Health, and many professional medical organizations.

Meanwhile, we should note that acceptance of research payments is associated with more favorable results and conclusion for the pharmaceutical companies sponsoring trials ^(5), (6)^. Pharmaceutical companies have made payments for meal and beverage, travel and accommodation fees, and speaking fees to market their products with less efficacy and sometimes less safety ^(7)^. We concluded that few physicians and HCPs received personal payments concerning the COVID-19 mRNA vaccines in the US. This study had several limitations: inaccuracies of the Open Payments Database and underestimation of financial relationships with industries uncovered by the Open Payments Database, as the product data relating to the payments before FDA approval timing of the mRNA vaccines were not included in the Open Payments Database.

## Article Information

### Conflicts of Interest

Dr. Saito received personal fees from Taiho Pharmaceutical Co. Ltd outside the scope of the submitted work. Drs. Ozaki and Tanimoto received personal fees from Medical Network Systems, a dispensing pharmacy, outside the scope of the submitted work. Dr. Tanimoto also received personal fees from Bionics Co. Ltd, a medical device company, outside the scope of the submitted work. Regarding nonfinancial conflicts of interest, all are engaged in ongoing research examining financial and nonfinancial conflicts of interest among healthcare professionals and pharmaceutical companies in Japan and the United States.

### Author Contributions

Anju Murayama: Data collection, study concept and design, statistical analysis, drafting of the manuscript, revising of the manuscript, and study administration

Sae Kamamoto: Data collection, study concept and design, statistical analysis, drafting of the manuscript, and revising of the manuscript

Hiroaki Saito: Study concept and design, critically reviewing of the manuscript, and revising of the manuscript

Tetsuya Tanimoto: Study concept and design, drafting of the manuscript, and study supervision

Akihiko Ozaki: Study concept and design, critically reviewing of the manuscript, revising of the manuscript, and study supervision

### Approval by Institutional Review Board (IRB)

This study was approved by the Ethics Committee of the Medical Governance Research Institute (approval number: MG2018-04-20200605; approval date: June 5, 2020). Informed consent was waived, as this study included only publicly available information.
